# Young Adult Substance Use as a Predictor of Poor Memory Decades Later in Midlife

**DOI:** 10.1093/geroni/igaf122.4050

**Published:** 2025-12-31

**Authors:** Yuk Pang, Yvonne Terry-McElrath, Joy Bohyun Jang, Megan Patrick

**Affiliations:** University of Michigan, Ann Arbor, Michigan, United States; University of Michigan, Ann Arbor, Michigan, United States; University of Michigan, Ann Arbor, Michigan, United States; University of Michigan, Ann Arbor, Michigan, United States

## Abstract

Subjective memory decline is an early sign of cognitive decline and dementia. Understanding risk factors associated with midlife poor memory is crucial for effective prevention. This study prospectively examines associations between young adult (YA) heavy substance use and late midlife poor memory, and the mediating role of early midlife problematic substance use. Data on number of waves of YA (age 18-30) heavy alcohol, binge drinking, cannabis and cigarette use, early midlife (age 35) substance use disorder symptoms and heavy cigarette use, and late midlife poor self-rated memory (age 50-65) were from the Monitoring the Future (MTF) Panel study (N = 14,659). Mediation analyses assessed whether early midlife problematic substance use mediated YA heavy substance use associations with late midlife poor memory. YA heavy alcohol use and binge drinking were associated with higher odds of early midlife disordered (vs. non-disordered) alcohol use (AOR=1.69 and 1.66, both p<.001), and with late midlife poor memory (AOR=1.34 and 1.39, both p<.001). Partial mediation for alcohol (AOR=1.17, p<.001) and full mediation for binge drinking (AOR=1.18, p<.001) were observed. YA heavy cannabis use was associated with early midlife disordered use (vs. non-disordered; AOR=1.42, p<.001), and with late midlife poor memory (AOR 1.41, p = 0.008); full mediation (AOR=1.13, p = 0.010) was observed. YA daily cigarette use was directly associated with late midlife poor memory (AOR=1.05, p = 0.047), with no evidence of mediation. These findings underscore long-term risks of sustained YA substance use for cognition, and indicate that long-term associations may partially or completely operate through early midlife disordered substance use.

